# Advancing clinical neuroscience in Romania: a decade of experience from the RoNeuro Institute for Neurological Research and Diagnostic

**DOI:** 10.25122/jml-2024-1005

**Published:** 2024-03

**Authors:** Dafin Fior Mureşanu

**Affiliations:** 1 RoNeuro Institute for Neurological Research and Diagnostic, Cluj-Napoca, Romania; 2 Department of Neuroscience, Iuliu Hatieganu University of Medicine and Pharmacy, Cluj-Napoca, Romania

Since its inception, The RoNeuro Institute, part of the Foundation for the Study of Nanoneurosciences and Neuroregeneration, was set to change the paradigm of clinical, educational, and research practices in Romania. As a founder, I have witnessed a decade of transformative advancements in neuroscience, laying the groundwork for a distinctive, private, non-profit model. This model seamlessly integrates clinical practice, education, and research within a multidisciplinary framework, empowering young healthcare professionals to remain in our country. Our aim has always been to elevate neurology and neuroscience at a national level, ultimately improving the neurological health of the Romanian population.

Over the years, the study of neurological diseases, including investigations into the therapeutic effects of pharmacological and non-pharmacological interventions and economic analyses, has translated into over 200 published and co-authored scientific articles. We have established strong partnerships with international societies, fostering a global network for collaborative research. We have made significant contributions to understanding conditions such as stroke and traumatic brain injury (TBI), which pose major public health challenges in Romania, contributing to high mortality and morbidity rates and imposing a substantial economic burden on our healthcare system [1].

Our research has delved into the therapeutic potential of pharmacological interventions for post-stroke neuroregeneration, and we have actively participated in developing European guidelines for motor rehabilitation after acute ischemic stroke [2-4].

The research team, which includes medical doctors and residents, psychologists, and public health specialists, has combined their efforts and expertise to bridge the gaps between research and clinical practice. Using innovative neurophysiological tools and neurotrophic agents, the research activity at the RoNeuro Institute aims to improve functional outcomes and quality of life for patients affected by neurological disorders. In the field of TBI, we have investigated biological markers, pharmacological agents like Cerebrolysin, and non-pharmacological rehabilitation strategies such as repetitive transcranial magnetic stimulation (rTMS) [5,6]. Our studies have demonstrated the positive impact of these interventions on patient recovery, opening new avenues for treatment [5, 6]. Furthermore, we have explored the concept of cognitive reserve in relation to chronic traumatic encephalopathy resulting from TBI, aiming to understand the brain's resilience to trauma [7].

The research interests of the RoNeuro Institute extend beyond stroke and TBI. We have conducted studies on Alzheimer's disease, seeking to enhance our understanding of this devastating neurodegenerative condition and improve treatment strategies [8,9]. Additionally, our work on post-traumatic stress disorder (PTSD) and associated comorbidities highlights our commitment to addressing the psychological consequences of trauma in clinical settings [10]. Recognizing the vital role of sleep in neurological and psychological health, we conducted the first national study on sleep quality in the Romanian population, providing valuable insights into sleep disorders and habits [11].

Our vision goes beyond merely improving functional outcomes after neurological injury. We are committed to enhancing brain capacity and optimizing neurological health and function. This commitment is reflected in our ongoing N-HANCE project, which evaluates the role of dietary supplementation on attention, cognition, and mental well-being in individuals experiencing subjective cognitive complaints.

The COVID-19 pandemic has had a profound impact on neurological health worldwide. In response, experts from the RoNeuro Institute collaborated with the European Academy of Neurology (EAN) to issue a call for action. This included the establishment of a multidisciplinary task force, an online neuroCOVID survey, and the EAN Neuro COVID-19 registry. These initiatives aimed to provide timely information and support to healthcare professionals and researchers during the pandemic [12].

**Figure 1:**
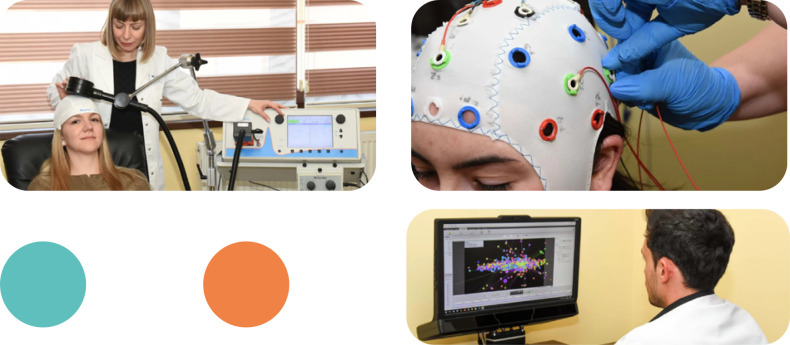
RoNeuro's rTMS (left), qEEG (top-right), and eye tracking (bottom-right) labs

The RoNeuro Institute is at the forefront of utilizing innovative neurotechnologies to advance neurological care. Our three specialized departments – the rTMS lab, the qEEG lab, and the Eye-tracking lab – are each equipped with state-of-the-art equipment and led by experts in their respective fields (Figure 1):

- rTMS Lab: This lab employs non-invasive brain stimulation to investigate brain function and connectivity. rTMS has applications in both diagnosis and therapy, aiding in the detection of motor abnormalities, assessment of cognitive processes, and treatment of conditions like chronic pain, depression, and movement disorders [13, 14].
- qEEG Lab: Quantitative EEG (qEEG) provides detailed insights into brain electrical activity, assisting in the diagnosis of conditions such as epilepsy, encephalopathy, dementia, and TBI [15-18]. Our qEEG lab also hosts the annual NeurotechEU Summer School of QEEG, training young doctors across Europe.
- Eye-Tracking Lab: By analyzing eye movements, our eye-tracking lab contributes to the evaluation of responses to pharmacological treatments and neurocognitive rehabilitation in various neurological, psychological, and psychiatric disorders, including stroke, TBI, chronic pain, and extrapyramidal disorders [19].

Looking ahead, the RoNeuro Institute remains dedicated to fostering research, embracing new technologies, and strengthening collaborations. We envision a future where our work continues to drive innovation, improve patient outcomes, and enhance the quality of life for individuals affected by neurological disorders.

The past decade has been a journey of overcoming challenges, celebrating successes, and recognizing the power of multidisciplinary teamwork. We are committed to building upon these lessons as we move forward, striving for excellence in neurological research and clinical care. Together, we can make a lasting impact on the lives of those we serve.
